# Achievement of long-term treatment goals in upadacitinib-treated patients with moderately to severely active ulcerative colitis: a post hoc analysis of phase 3 trial data

**DOI:** 10.1093/ecco-jcc/jjaf095

**Published:** 2025-06-09

**Authors:** Julian Panés, Marla C Dubinsky, Yoh Ishiguro, Nidhi Shukla, Elena Dubcenco, Valencia Remple, Dolly Sharma, Remo Panaccione

**Affiliations:** Hospital Clinic Barcelona, IDIPABS, CIBERehd, Barcelona, Spain; Susan and Leonard Feinstein IBD Center, Icahn School of Medicine, Mount Sinai, New York, NY, United States; Department of Clinical Research, Hirosaki General Medical Center, National Hospital Organization, Hirosaki, Japan; AbbVie Inc., Chicago, IL, United States; AbbVie Inc., Chicago, IL, United States; AbbVie Inc., Chicago, IL, United States; AbbVie Inc., Chicago, IL, United States; Department of Gastroenterology and Hepatology, University of Calgary, Calgary, AB, Canada

**Keywords:** clinical trials, quality of life, socio-economical and psychological endpoints

## Abstract

**Background and Aims:**

Comprehensive disease control is an important yet elusive treatment goal in ulcerative colitis (UC). We evaluated the effect of upadacitinib induction and maintenance treatment on composite clinical endpoints and normalization of important health-related quality of life (HRQoL) outcomes.

**Methods:**

Data from the U-ACHIEVE and U-ACCOMPLISH trials were analyzed. Clinical responders to upadacitinib 45 mg 8-week induction were re-randomized 1:1:1 to upadacitinib 30 mg, 15 mg, or placebo for 52 weeks of maintenance. The percentage of patients achieving a composite clinical endpoint (clinical remission, complete symptom resolution, and Inflammatory Bowel Disease Questionnaire [IBDQ] remission) and a composite endpoint for normalization of HRQoL outcomes (Functional Assessment of Chronic Illness Therapy-Fatigue, IBDQ remission, Work Productivity and Activity Impairment Questionnaire-Ulcerative Colitis, 36-item Short Form Survey Physical Component Summary and Mental Component Survey, and EuroQol 5-Dimension 5-Level scores) was evaluated.

**Results:**

At induction week 8, more patients treated with upadacitinib 45 mg achieved the composite clinical endpoint vs placebo (6.4% vs 0.9%, *P* ≤ .001) and normalization of the composite HRQoL endpoint (18.9% vs 5.5%, *P* ≤ .001). At maintenance week 52, the composite clinical endpoint was achieved by 18.3% and 13.1% of patients treated with upadacitinib 30 mg and 15 mg, respectively, vs 4.5% with placebo (*P* ≤ .001). Normalization of the composite HRQoL endpoint was achieved by 24.0% and 22.3% of patients treated with upadacitinib 30 mg and 15 mg, respectively, vs 8.7% for placebo (*P* ≤ .001).

**Conclusions:**

Upadacitinib may help patients with moderately to severely active UC achieve complete symptom resolution, endoscopic remission, and normalization of HRQoL.

**Clinical registration numbers:**

U-ACHIEVE (NCT02819635) and U-ACCOMPLISH (NCT03653026)

## Graphical Abstract

**Graphical Abstract:**
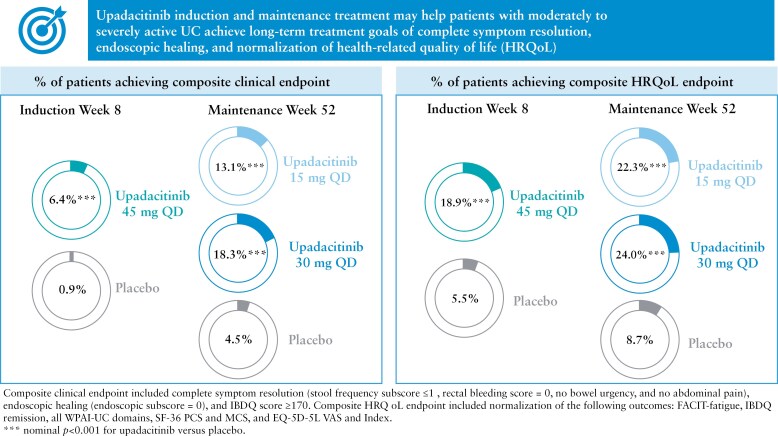

## 1. Introduction

Ulcerative colitis (UC) symptoms such as abdominal pain, bowel urgency, stool frequency, and rectal bleeding can be debilitating and negatively affect a patient’s quality of life.^1–4^ Per the Selecting Therapeutic Targets in Inflammatory Bowel Disease II initiative, short-term treatment goals in UC include reduction in symptoms and inflammatory biomarkers, while clinical remission, endoscopic healing, and normalization of quality of life are considered long-term goals.^5^

Results from the Phase 3 clinical program, which included the upadacitinib induction and maintenance trials, U-ACHIEVE and U-ACCOMPLISH, have shown that patients with moderately to severely active UC who were treated with upadacitinib had significant improvements in the individual outcomes of stool frequency, rectal bleeding, bowel urgency, abdominal pain, endoscopic remission, and Inflammatory Bowel Disease Questionnaire (IBDQ) remission.^6–9^ However, there is a need to better understand the extent to which patients treated with upadacitinib can achieve composite clinical and health-related quality of life (HRQoL) endpoints, which are more stringent than stand-alone endpoints in the short- or long-term. Therefore, this study aimed to examine the extent to which patients with moderately to severely active UC who were treated with upadacitinib can achieve a stringent composite clinical endpoint and normalization of a composite HRQoL endpoint at the end of induction and after 1 year of maintenance therapy.

## 2. Materials and methods

### 2.1 Study population

In the U-ACHIEVE (NCT02819635) and U-ACCOMPLISH (NCT03653026) studies, patients with a clinical response after 8-week induction treatment with upadacitinib 45 mg once daily (QD) were enrolled in the maintenance trial and re-randomized 1:1:1 to upadacitinib 15 mg QD, upadacitinib 30 mg QD, or placebo QD.^7,10^ Approximately half of the patients enrolled in these studies had a history of inadequate response to at least one biologic treatment (bio-IR). Full details of the eligibility criteria and primary efficacy and safety results for the U-ACHIEVE and U-ACCOMPLISH studies have been published elsewhere.^7,10^ These studies were conducted in accordance with the International Conference on Harmonisation guidelines and the Declaration of Helsinki. The protocol was approved by an institutional review board or independent ethics committee at each site. Written informed consent was provided by all patients before screening.

### 2.2 Outcomes

Attainment of treatment goals was determined based on the percentage of patients who achieved the composite clinical endpoint of endoscopic remission, complete symptom resolution, and IBDQ remission. Patients who achieved the composite clinical endpoint had to have an endoscopic subscore = 0, stool frequency subscore ≤ 1, rectal bleeding subscore = 0, no bowel urgency, no abdominal pain, and an IBDQ total score ≥ 170. The percentage of patients who achieved the composite clinical endpoint was assessed at the end of induction (week 8) and at maintenance week 52.

To better understand the benefits of upadacitinib treatment across varying patient populations, we also examined the percentages of patients who achieved the composite clinical endpoint after induction and 1 year of maintenance therapy with upadacitinib stratified by prior biologic use, disease severity, and disease duration. Finally, we evaluated the extent to which patients treated with upadacitinib achieved normalization of HRQoL outcomes after induction and maintenance treatment. The percentage of patients achieving normalization of each HRQoL outcome was based on literature-normative values assessed at induction week 8 and maintenance week 52. Normative thresholds for each outcome were as follows: Functional Assessment of Chronic Illness Therapy-Fatigue (FACIT-Fatigue) ≥ 40.1,^11^ IBDQ remission score ≥ 170,^12^ Work Productivity and Activity Impairment Questionnaire: Ulcerative Colitis (WPAI-UC) 0% for each component (overall work impairment, work time missed, impairment while working, daily activity impairment), 36-item Short Form Survey (SF-36) Physical Component Summary (PCS) score ≥ 50,^13–15^ SF-36 Mental Component Summary (MCS) score ≥ 50,^13–15^ EuroQol 5-Dimension 5-Level (EQ-5D-5L) visual analog scale (VAS) ≥ 80,^16^ EQ-5D-5L Index ≥ 0.825,^16^ and the composite HRQoL endpoint: normalization of all HRQoL outcomes.

### 2.3 Data analysis

Induction results were based on data collected from 988 patients (placebo, *n = *328; upadacitinib 45 mg QD, *n = *660) in the U-ACHIEVE and U-ACCOMPLISH studies. At induction week 8, the adjusted response rate between placebo and upadacitinib 45 mg was calculated using the Cochran-Mantel-Haenszel (CMH) test, adjusting for induction baseline corticosteroid use (yes or no), Adapted Mayo score (≤ 7 or > 7), and inadequate response to biologic (bio-IR) status (bio-IR or non-bio-IR).

Maintenance results were based on data collected from 681 patients who achieved a clinical response per Adapted Mayo score after 8-week induction treatment with upadacitinib 45 mg in the U-ACHIEVE and U-ACCOMPLISH studies and were re-randomized 1:1:1 to upadacitinib 15 mg QD (*n = *225), upadacitinib 30 mg QD (*n = *233), or placebo QD (*n = *223).^10^ Clinical response was defined as a decrease in Partial Mayo score ≥ 2 points and ≥ 30% from baseline, plus a decrease in rectal bleeding score ≥ 1 or an absolute rectal bleeding score ≤ 1.^9^ Only 8-week clinical responders to upadacitinib were included in the analysis of maintenance results. The adjusted response rate between treatment groups in patients who achieved the composite clinical endpoint at maintenance weeks 0 and 52 was calculated using the CMH test, adjusting for bio-IR status (bio-IR or non-bio-IR) at induction baseline, clinical remission status at maintenance week 0, and corticosteroid use at maintenance week 0.

We also assessed the proportion of patients who achieved complete symptom resolution at induction week 8 and maintenance week 52 stratified by the number of biologics used in the past (0, 1, or 2+), disease severity where moderate was defined as Adapted Mayo score 5 to ≤ 7 vs severe which was defined as Adapted Mayo score > 7, and disease duration (by year quartiles). Quartiles were defined as follows: Q1 (0 to 2.7 years), Q2 (2.7 to 5.8 years), Q3 (5.8 to 11.2 years), and Q4 (> 11.2 years). Adjusted differences between treatment groups (with 95% CI and *P*-values) were compared using the CMH test. Adjustments were made for corticosteroid use (yes or no) at induction baseline, Adapted Mayo score (≤ 7 or > 7) at induction baseline, and bio-IR status (bio-IR or non-bio-IR) at induction baseline. Calculations were based on non-responder imputation incorporating multiple imputations to handle missing data due to COVID-19 or non-responder imputation if there were no missing data due to COVID-19.

Health-related quality of life outcomes were reported for a subset of patients in the prespecified primary analysis population^7^ comprising the first 451 upadacitinib 45 mg QD 8-week induction responders who were enrolled in the 52-week maintenance study. Cohort sizes were upadacitinib 15 mg QD (*n = *148), upadacitinib 30 mg QD (*n = *154), and placebo QD (*n = *149). The percentage of patients achieving normalization of each of the following HRQoL outcomes, FACIT-Fatigue score, IBDQ remission score, SF-36 PCS, SF-36 MCS, EQ-5D-5L VAS, and EQ-5D-5L index, was examined at induction week 8 and maintenance week 52. A stringent composite score that included normalization of all HRQoL outcomes was also calculated at induction week 8 and maintenance week 52. Point estimates were determined for individual and composite scores, and treatment differences between upadacitinib and placebo were compared using the CMH test.

## 3. Results

### 3.1 Achievement of the composite clinical endpoint of endoscopic remission, complete symptom resolution, and IBDQ remission

**Figure 1.**
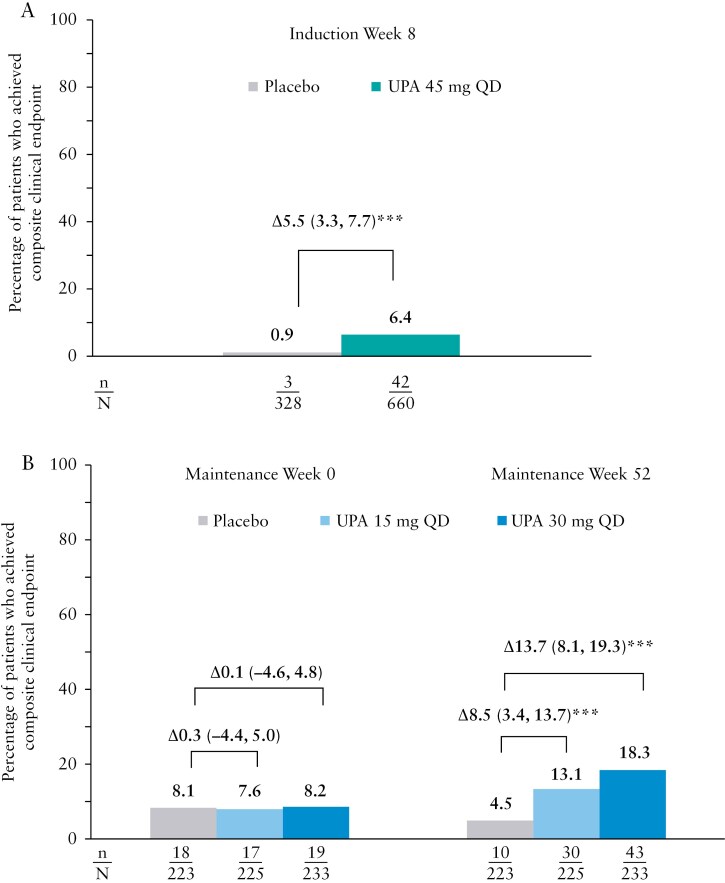
Percentage of patients who achieved the composite endpoint of endoscopic remission, complete symptom resolution, and IBDQ remission at (A) induction week 8 and (B) maintenance weeks 0 and 52. ***p ≤ 0.001 for upadacitinib (UPA) versus placebo (PBO).

A total of 988 patients (660 in the upadacitinib 45 mg QD group and 328 in the placebo group) participated in the phase 3 induction studies. The baseline characteristics of this patient population were similar across treatment groups and have been described in detail in the U-ACHIEVE and U-ACCOMPLISH studies, which have been published elsewhere.^7,10^ At induction week 8, a significantly greater percentage of patients treated with upadacitinib 45 mg QD achieved endoscopic remission, complete symptom resolution, and IBDQ remission vs placebo (6.4% vs 0.9%, *P* ≤ .001, Figure 1A).

A total of 681 of 988 patients were 8-week clinical responders to upadacitinib 45 mg induction treatment and were randomly assigned (1:1:1) to upadacitinib 15 mg QD (*n = *225), upadacitinib 30 mg QD (*n = *233), or placebo (*n = *223) in the maintenance study. Details of patient disposition for the U-ACHIEVE maintenance study have been published elsewhere.^10^ At maintenance week 52, the percentage of induction responders who achieved the composite clinical endpoint reached 18.3% with upadacitinib 30 mg and 13.1% with upadacitinib 15 mg vs 4.5% with placebo (*P* ≤ .001, Figure 1B). The number of induction responders who achieved the composite clinical endpoint increased between week 8 and week 52 in patients receiving upadacitinib maintenance, but not in the placebo maintenance group.

**Figure 2.**
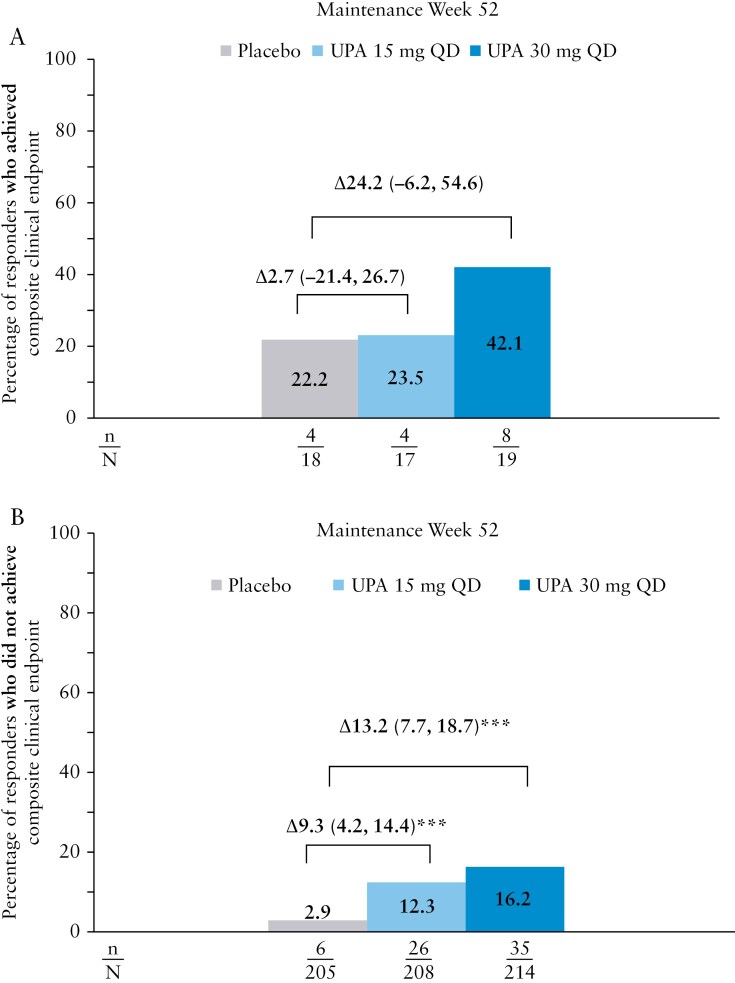
(A) Percentage of patients who achieved the composite clinical endpoint of endoscopic remission, complete symptom resolution, and IBDQ remission at maintenance week 52 among those *who achieved* the composite clinical endpoint at maintenance week 0. (B) Percentage of patients who achieved the composite clinical endpoint of endoscopic remission, complete symptom resolution, and IBDQ remission at maintenance week 52 among those who did not achieve the composite clinical endpoint at maintenance week 0. ***p ≤ 0.001 for upadacitinib (UPA) versus placebo (PBO).

Of the patients who achieved the composite clinical endpoint at week 0 of maintenance, 42.1%, 23.5%, and 22.2% in the upadacitinib 30 mg, upadacitinib 15 mg, and placebo groups, respectively, sustained the stringent endpoint at week 52 (Figure 2A). Among patients who did not achieve the composite clinical endpoint at week 0 of maintenance, 16.2%, 12.3%, and 2.9% in the upadacitinib 30 mg, upadacitinib 15 mg, and placebo groups, respectively, achieved it at week 52 (Figure 2B).

### 3.2 Achievement of the composite clinical endpoint of endoscopic remission, complete symptom resolution, and IBDQ remission stratified by prior biologic treatment, disease severity, and disease duration

**Figure 3.**
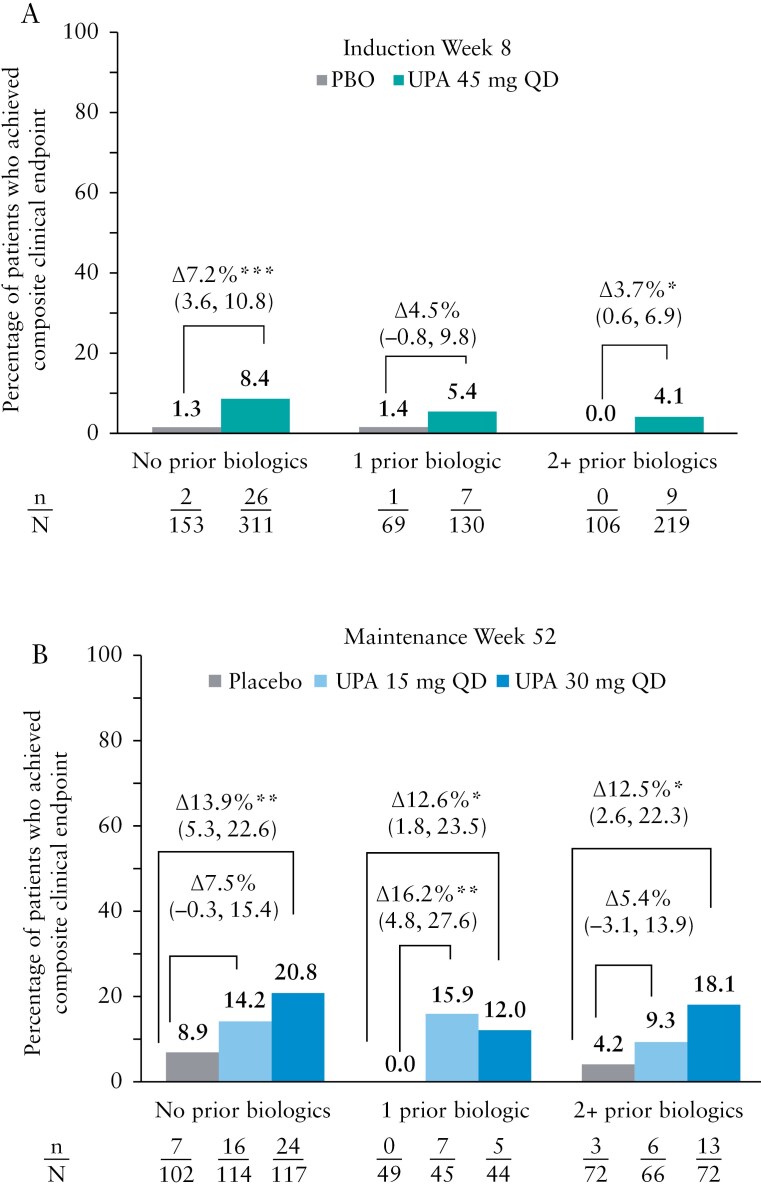
Percentage of patients who achieved the composite clinical endpoint of endoscopic remission, complete symptom resolution, and IBDQ remission at (A) induction week 8 and (B) maintenance week 52 stratified by prior biologic use at induction baseline. *p ≤ 0.05, **p ≤ 0.01, ***p ≤ 0.001 for upadacitinib (UPA) versus placebo (PBO)..

Of the 988 patients participating in the induction studies, 47% (464/988) were biologic-naïve, 20% (199/988) received previous treatment with 1 biologic, and 33% (325/988) received previous treatment with ≥ 2 biologics. Across the subgroups stratified by prior biologic use, a numerically greater percentage of patients treated with upadacitinib 45 mg vs placebo achieved the composite clinical endpoint at induction week 8 (no prior biologics: 8.4% vs 1.3%, *P* < .001; 1 prior biologic: 5.4% vs 1.4%, *P* = not significant [NS]; 2 + prior biologics: 4.1% vs 0%, *P* ≤ .5, Figure 3A). A similar trend was observed at week 52, with more patients treated with upadacitinib 30 mg and 15 mg vs placebo achieving the composite clinical endpoint regardless of prior biologic exposure status (no prior biologics: 20.8% and 14.2% vs 6.9%, *P* = NS and *P* ≤ .01, respectively; 1 prior biologic: 12.0% and 15.9% vs 0%, *P* ≤ .01 and *P* ≤ .05, respectively; 2 + prior biologics: 18.1% and 9.3% vs 4.2%, *P* = NS and *P* ≤ .05, respectively) (Figure 3B).

At induction baseline, 61% (599/988) had moderate disease and 39% (389/988) had severe disease. When data were stratified by disease severity, a greater percentage of patients treated with upadacitinib 45 mg vs placebo achieved the composite clinical endpoint at induction week 8 (moderate disease: 6.5% vs 1.0%, respectively, *P* < .001; severe disease: 6.2% vs 0.8%, respectively, *P* ≤ .01). At week 52, more patients achieved the composite endpoint with upadacitinib 30 mg and 15 mg vs placebo (moderate disease: 17.7% and 13.7% vs 3.8%, *P* < .001 and *P* ≤ .01, respectively; severe disease: 19.2% and 12.1% vs 5.4%, *P* ≤ .01 and *P* = NS, respectively) (Figure S1A and B).

Disease duration was assessed by 4-year quartiles, which were defined as Q1 (0–2.7 years), Q2 (2.7–5.8 years), Q3 (5.8–11.2 years), and Q4 (> 11.7 years). The following trend was observed when data were stratified by disease duration: greater percentages of patients treated with upadacitinib compared with placebo achieved the composite clinical endpoint at both induction week 8 and maintenance week 52, regardless of how long they had UC (Figure S2).

### 3.3 Achievement of normalization of HRQoL outcomes with upadacitinib treatment

We examined the proportion of patients achieving normalization of HRQoL outcomes in 451 upadacitinib 8-week clinical responders (upadacitinib 15 mg QD, *n = *148; upadacitinib 30 mg QD, *n = *154; and placebo QD, *n = *149). As shown in Figure S3A and B, significantly more patients achieved normalization of FACIT-Fatigue and IBDQ remission scores with upadacitinib than placebo at induction week 8 and maintenance week 52 (Figure S4A and B). Similar results were observed in patients in the upadacitinib groups vs placebo for SF-36 PCS, SF-36-MCS, EQ-5D-5L VAS, and EQ-5D-5L Index at both induction week 8 and maintenance week 52 (Figure S4A and B). Significantly more patients achieved normalization of WPAI-UC domain scores including overall work impairment, work time missed, impairment while working, and daily activity impairment with upadacitinib treatment compared with placebo at induction week 8 and maintenance week 52 (Figure S5A and B).

**Figure 4.**
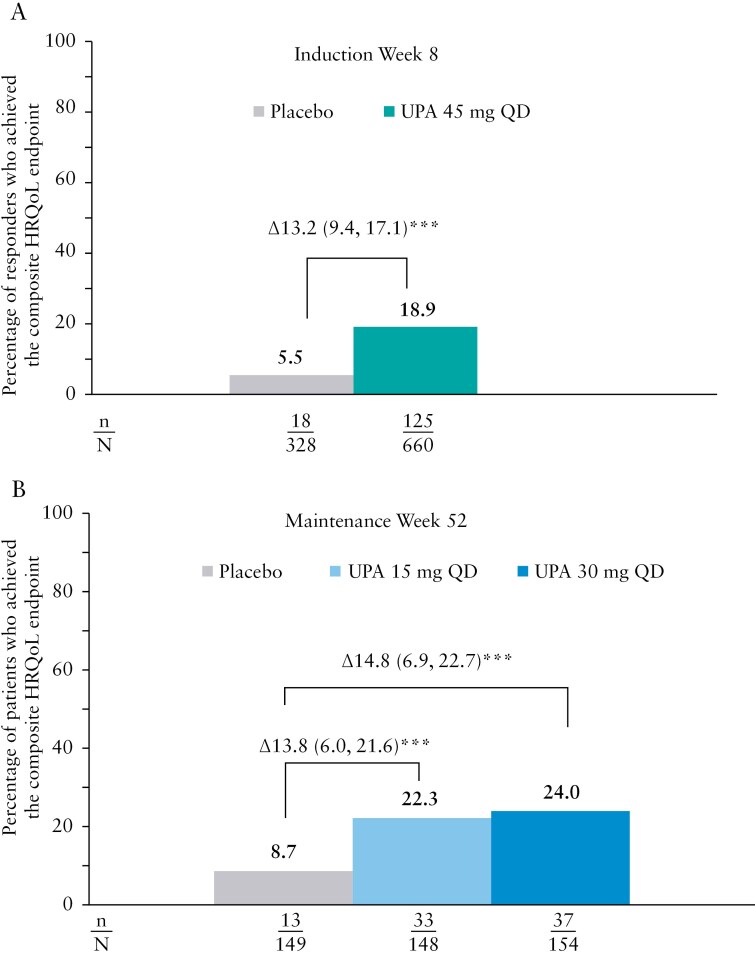
Percentage of patients achieving normalization of the composite HRQoL endpoint with upadacitinib treatment at (A) induction week 8 and (B) maintenance week 52. ***p ≤ 0.001 for upadacitinib (UPA) versus placebo (PBO).

We also examined the effect of upadacitinib vs placebo on achievement of a composite HRQoL endpoint, which included normalization of all the HRQoL outcomes (FACIT-Fatigue, IBDQ remission, all WPAI-UC domains [overall work impairment, work time missed, impairment while working, daily activity impairment]), SF-36 PCS, SF-36 MCS, EQ-5D-5L VAS, and EQ-5D-5L Index. This analysis showed that significantly more patients achieved normalization of the composite HRQoL endpoint with upadacitinib compared with placebo at induction week 8 (18.9% vs 5.5%, *P* < .001, Figure 4A); and maintenance week 52 (upadacitinib 30 mg and 15 mg vs placebo, 24.0% and 22.3% vs 8.7%, both *P* < .001, respectively (Figure 4B).

## 4. Discussion

Treat-to-target strategies have been identified for adults with moderately to severely active UC. Short-term treatment goals of managing symptoms and reducing inflammation while long-term goals include achieving normalization of quality of life and endoscopic healing.^5^ These treatment goals are important because symptom control results in less impact of UC on a patient’s daily and professional life, while reducing inflammation and promoting endoscopic healing reduces the number of UC-related flares and the risk of bowel damage. Upadacitinib has been shown to provide rapid and significant improvement in UC symptoms including bowel urgency, abdominal pain, stool frequency, and rectal bleeding, and mucosal healing individually.^6–9^ In this study, we assessed the extent to which patients treated with upadacitinib achieve a stringent, clinically relevant, composite endpoint of endoscopic remission (defined as endoscopic subscore ≤ 1), complete symptom resolution (defined as stool frequency subscore ≤ 1, rectal bleeding subscore = 0, no bowel urgency, and no abdominal pain) as well as IBDQ remission (defined as total score ≥ 170) at the end of induction treatment and at 1 year of maintenance treatment. Attainment of the composite clinical endpoint indicates that the patient would have no ulceration of the intestinal mucosa, relatively normal bowel frequency, absence of rectal bleeding, a lack of bowel urgency, no abdominal pain, and achievement of IBDQ remission, suggesting that UC-specific symptoms are no longer impacting patients’ daily life. In addition, UC can impact social, emotional, psychological, and professional aspects of a patient’s life.^17–23^ Because a long-term treatment goal for UC is the restoration of quality of life, we also evaluated the extent to which patients achieved normalization of a composite HRQoL outcome score after upadacitinib induction and maintenance treatment. The composite HRQoL endpoint included disease-specific (IBDQ remission), general (SF-36-PCS, SF-36 MCS, and EQ-5D-5L) HRQoL measure and fatigue assessments as well as the impact on work productivity (including overall work impairment, work time missed, and impairment while working) and daily activity. Achievement of this complex composite HRQoL endpoint goes beyond symptom control and demonstrates a return to normal quality of life with substantial improvements across several areas that are relevant to patients such as reducing disease-related fatigue, improving physical and mental functioning, and being able to have a normal work life and perform daily activities. Of note, it has been reported in the literature that some patients still have persistent symptoms even if they achieve endoscopic remission and mucosal healing, suggesting that disease activity and symptoms may not always be related.^24^ These observations highlight the complexity of UC management and the importance of assessing composite endpoints beyond the individual endpoints commonly assessed in clinical trials.

Results of our post hoc analysis indicate that the rigorous, long-term treatment goals of endoscopic remission, complete symptom resolution, and IBDQ remission can be achieved with upadacitinib treatment in patients with moderately to severely active UC as early as week 8 of induction. Maintenance treatment with upadacitinib after induction treatment resulted in an increased proportion of patients achieving or sustaining these stringent long-term treatment goals after 1 year. At maintenance week 52, nearly 1 in 5 patients treated with upadacitinib 30 mg and approximately 1 in 8 patients treated with upadacitinib 15 mg had achieved or sustained this stringent target.

More upadacitinib-treated patients compared with placebo achieved the stringent composite clinical endpoint irrespective of prior biologic therapy, disease severity, and disease duration. Upadacitinib was also more effective than placebo in achieving normalization of HRQoL at both the end of induction and the end of the 52-week maintenance period, suggesting that upadacitinib may help patients with moderately to severely active UC attain the long-term treatment goal of restoration of quality of life.

Strengths of this post hoc analysis include the examination of a clinically relevant, stringent, composite clinical endpoint that assessed endoscopic remission, complete symptom resolution, and IBDQ remission as well as a composite endpoint of normalization of a range of HRQoL outcomes that included FACIT-Fatigue, SF-36 PCS and MCS, EQ-5D-5L, and WPAI-UC scores. Comprehensive endpoints such as these are not routinely examined in drug development programs, making this analysis a unique and clinically relevant approach to assessing restoration of quality of life in patients with UC. Data pooled from 2 phase 3 clinical trials provided a large sample size and the placebo-controlled nature of the study reduces potential bias associated with knowledge of receiving active treatment. A limitation of this analysis is its post hoc nature; therefore, results should be interpreted in this context. Also, because these data were obtained from a clinical trial setting with specific inclusion/exclusion criteria, the findings may not be generalizable to patients with mild UC or to patients in clinical practice. Also, the definitions of the clinical outcomes are based on the definitions reported in the phase 3 clinical trials and may not be readily applicable in clinical practice. Future studies investigating the maintenance of these clinical and HRQoL composite endpoints beyond 1 year are warranted. It is important to note that although this study focused on efficacy data, treatment decisions need to consider not only on the potential efficacy outcome, but also the individual patient’s risk of serious infection, malignancy, major adverse cardiovascular events, and thrombosis.

Results from this post hoc analysis demonstrate that upadacitinib may provide patients who have moderately to severely active UC with a therapeutic treatment option that results in the achievement of stringent composite clinical treatment goals of complete symptom resolution, endoscopic remission, and achievement of normalization of quality of life as early as week 8 of initiating induction treatment, which was further maintained long-term after 1 year of maintenance treatment.

## Supplementary Material

jjaf095_suppl_Supplementary_Figures_S1-S2

## Data Availability

AbbVie is committed to responsible data sharing regarding the clinical trials we sponsor. This includes access to anonymized, individual, and trial-level data [analysis datasets], as well as other information [eg, protocols, clinical study reports, or analysis plans], as long as the trials are not part of an ongoing or planned regulatory submission. This includes requests for clinical trial data for unlicensed products and indications. These clinical trial data can be requested by any qualified researchers who engage in rigorous, independent, scientific research, and will be provided following review and approval of a research proposal, statistical analysis plan, and execution of a data sharing agreement. Data requests can be submitted at any time after approval in the USA and Europe and after acceptance of this manuscript for publication. The data will be accessible for 12 months, with possible extensions considered. For more information on the process or to submit a request, visit the following link: [https://vivli.org/ourmember/abbvie/] then select “Home.”
