# Supplementary material for: Achievement of long-term treatment goals in upadacitinib-treated patients with moderately to severely active ulcerative colitis: a post hoc analysis of phase 3 trial data
Source: J Crohns Colitis. 2025 Jun 9;19(7):jjaf095. doi: 10.1093/ecco-jcc/jjaf095 (PMC12260494; doi:10.1093/ecco-jcc/jjaf095)

Achievement of long-term treatment goals in upadacitinib-treated patients with moderately to severely active ulcerative colitis: a post hoc analysis of phase 3 trial data

Julian Panés[1], Marla C Dubinsky[2], Yoh Ishiguro[3], Nidhi Shukla[4], Elena Dubcenco[4], Valencia Remple[4], Dolly Sharma[4], Remo Panaccione[5]

[1]Hospital Clinic Barcelona, IDIPABS, CIBERehd, Barcelona, Spain; [2]Susan and Leonard Feinstein IBD Center, Icahn School of Medicine, Mount Sinai, New York, NY, USA; [3]Department of Clinical Research, Hirosaki General Medical Center, National Hospital Organization, Hirosaki, Japan; [4]AbbVie Inc., Chicago, IL, USA; [5]Department of Gastroenterology and Hepatology, University of Calgary, Calgary, AB, Canada

SUPPLEMENTARY DATA

Supplementary Figure 1. Percentage of patients who achieved the composite clinical endpoint of endoscopic remission, complete symptom resolution, and IBDQ remission stratified by disease severity at induction baseline. Adjusted differences (with 95% CI and p-values) between treatment groups were compared using the Cochran-Mantel-Haenszel test adjusting for corticosteroid use (yes or no) at induction baseline, Adapted Mayo score (≤ 7 or > 7) at induction baseline, and bio-IR status (bio-IR or non-bio-IR) at induction baseline. Calculations were based on non-responder imputation incorporating multiple imputation to handle missing data due to COVID-19. *$p \leq 0.05$, **$p \leq 0.01$, ***$p < 0.001$ for upadacitinib versus placebo.

bio-IR, biologic inadequate responder; CI, confidence interval; IBDQ, Inflammatory Bowel Disease Questionnaire.

Supplementary Figure 1.

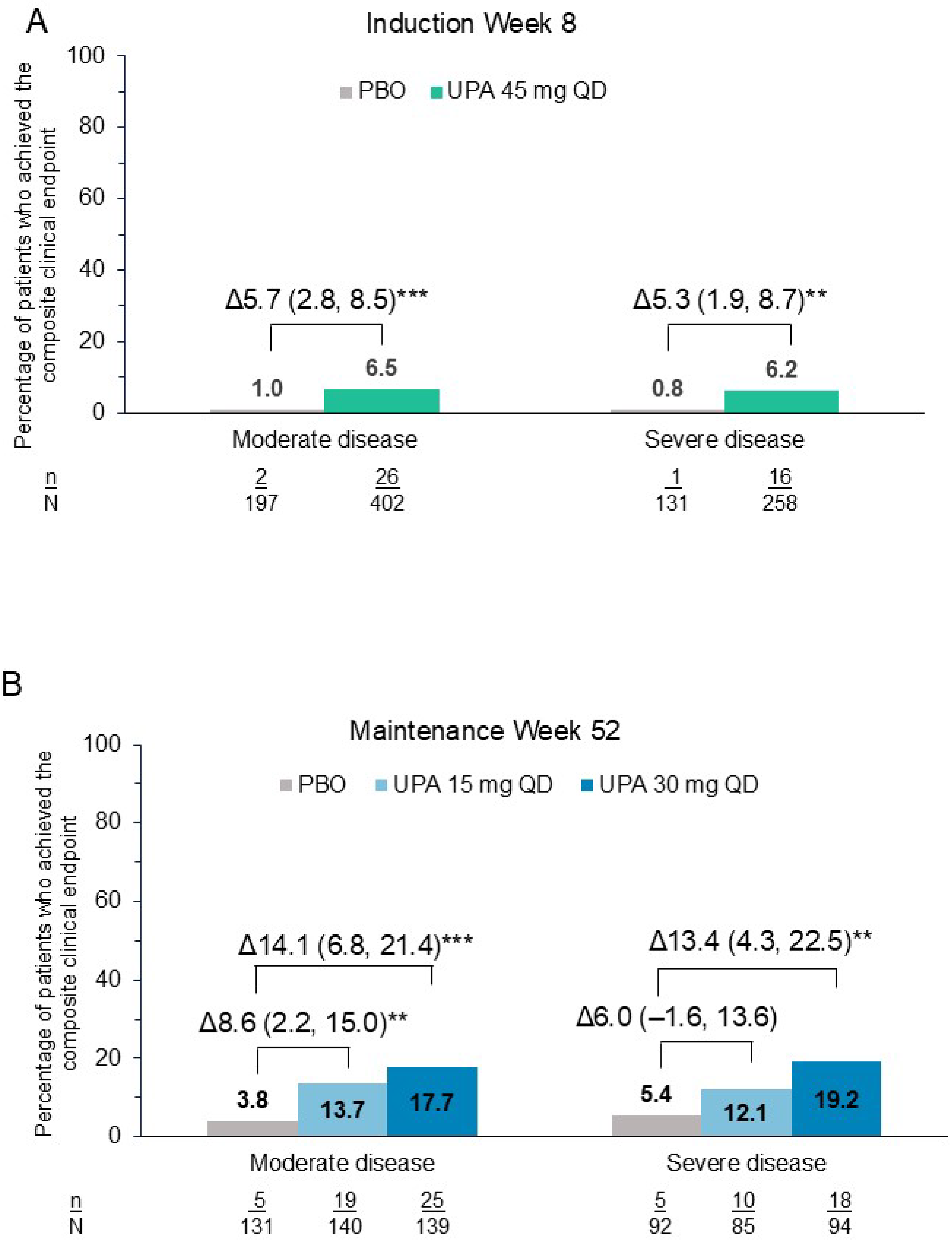

Supplementary Figure 2. Percentage of patients who achieved the composite clinical endpoint of endoscopic remission, complete symptom resolution, and IBDQ remission at week 8 and 52 stratified by disease duration at induction baseline. Adjusted differences (with 95% CI and p-values) between treatment groups were compared using the Cochran-Mantel-Haenszel test adjusting for corticosteroid use (yes or no) at induction baseline, Adapted Mayo score (≤ 7 or > 7) at induction baseline, and bio-IR status (bio-IR or non-bio-IR) at induction baseline. Calculations were based on non-responder imputation incorporating multiple imputation to handle missing data due to COVID-19. *$p \leq 0.05$, **$p \leq 0.01$, ***$p < 0.001$ for upadacitinib versus placebo.

bio-IR, biologic inadequate responder; CI, confidence interval; IBDQ, Inflammatory Bowel Disease Questionnaire.

Supplementary Figure 2.

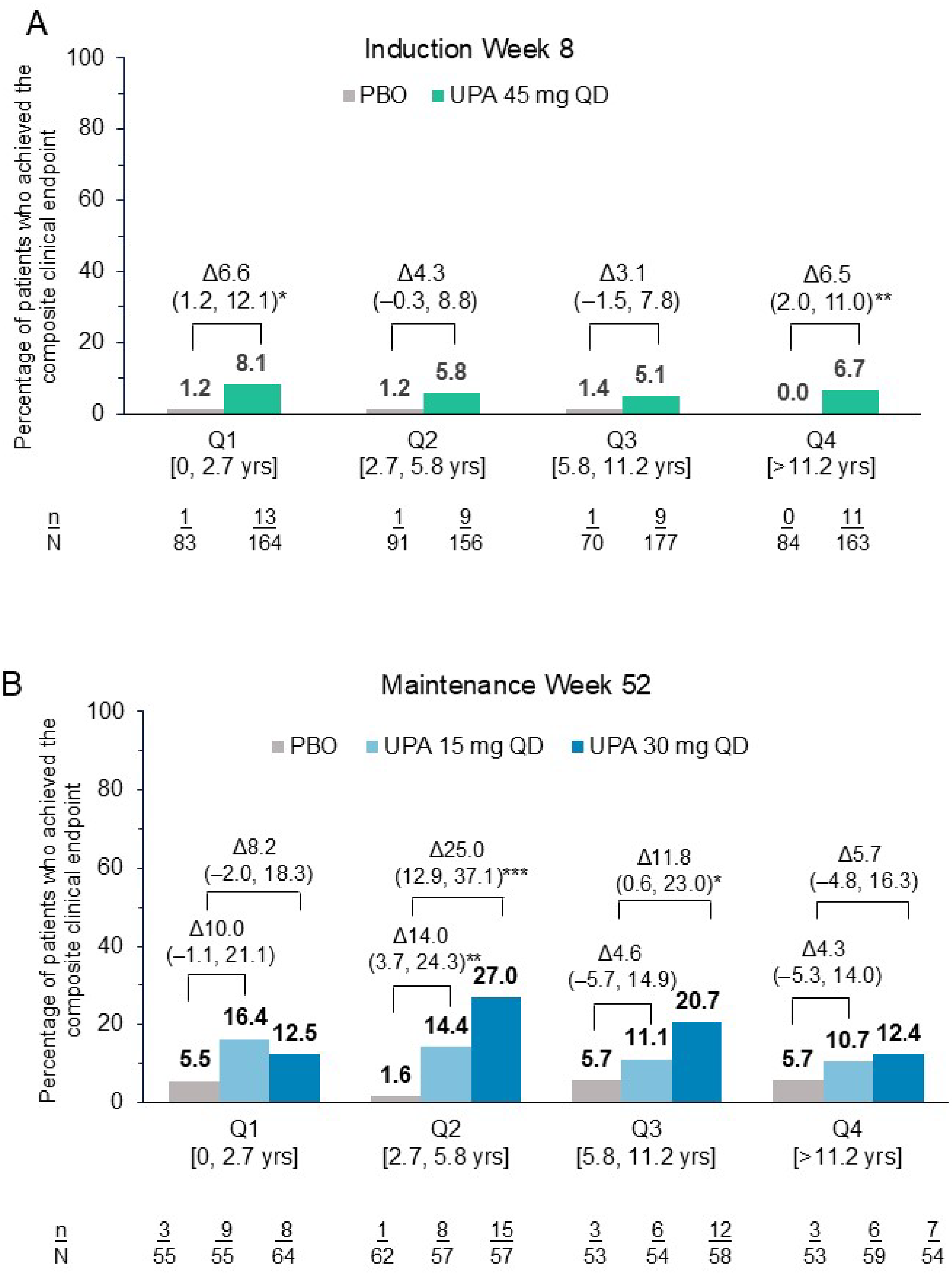

Supplementary Figure 3. Percentage of patients achieving normalization of FACIT-Fatigue (FACIT-F) and IBDQ scores with upadacitinib treatment at (A) induction week 8 and (B) maintenance week 52. *$p \leq 0.05$, **$p \leq 0.01$, ***$p \leq 0.001$ for upadacitinib (UPA) versus placebo (PBO).

Supplementary Figure 3.

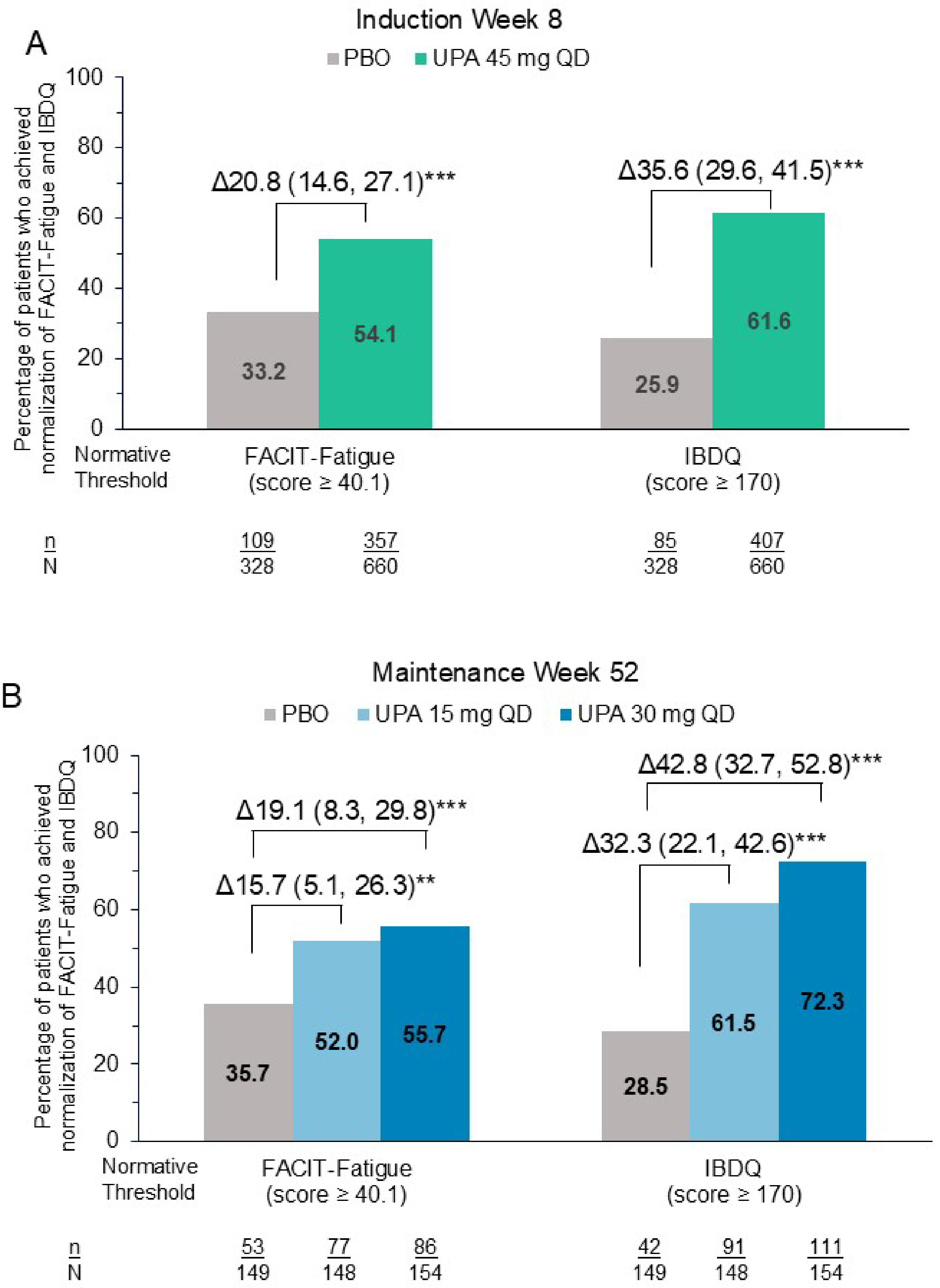

Supplementary Figure 4. Percentage of patients achieving normalization of SF-36 and EQ-5D-5L scores with upadacitinib treatment at (A) induction week 8 and (B) maintenance week 52. *$p \leq 0.05$, **$p \leq 0.01$, ***$p \leq 0.001$ for upadacitinib (UPA) versus placebo (PBO).

Supplementary Figure 4.

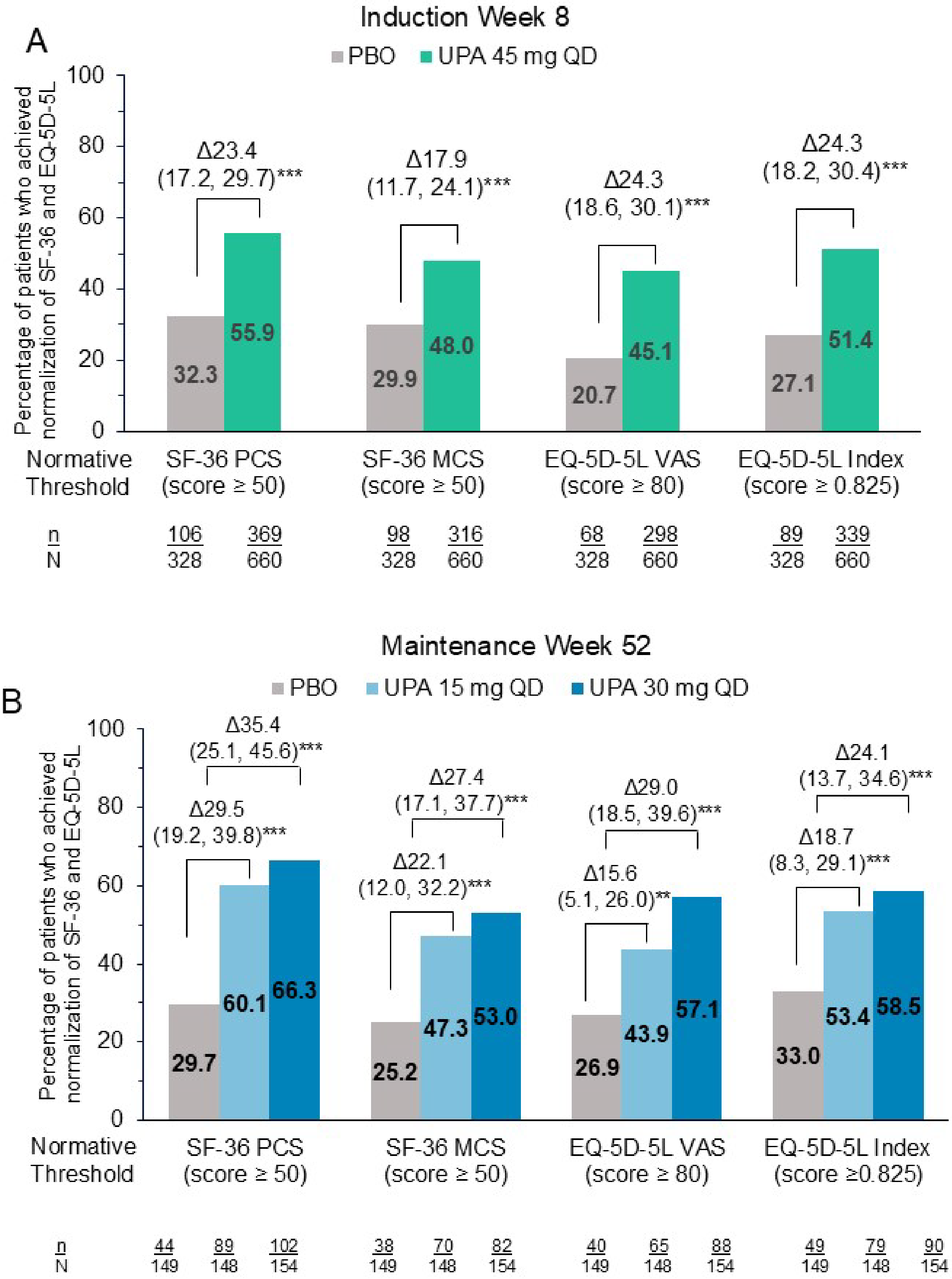

Supplementary Figure 5. Percentage of patients achieving normalization of all WPAI-UC scores with upadacitinib treatment at (A) induction week 8 and (B) maintenance week 52. *$p \leq 0.05$, **$p \leq 0.01$, ***$p \leq 0.001$ for upadacitinib (UPA) versus placebo (PBO).

Supplementary Figure 5.

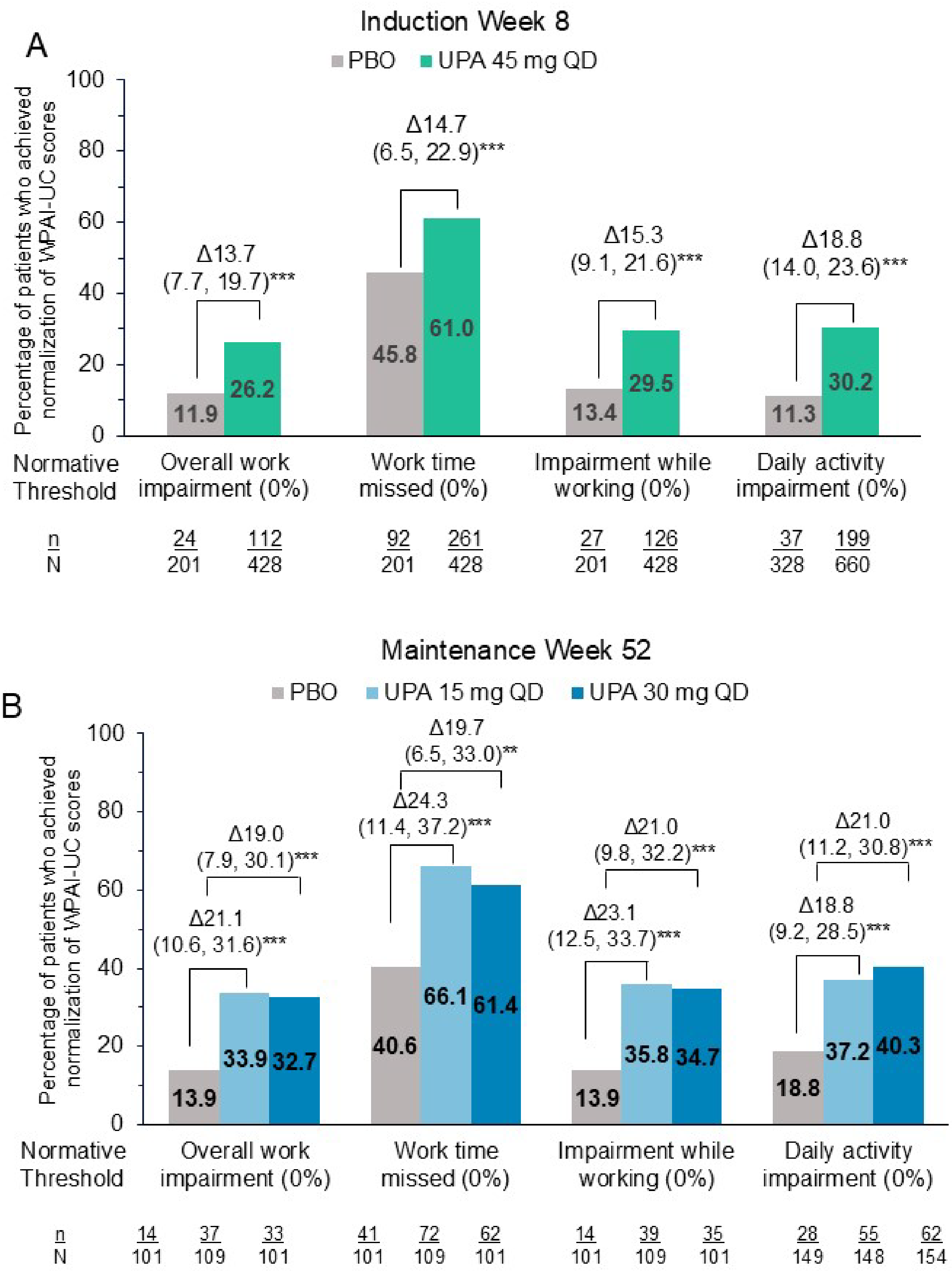

Supplement: jjaf095_suppl_Supplementary_Figures_S1-S2 [file jjaf095_suppl_supplementary_figures_s1-s2.pdf]
